# Race, Socioeconomic Status, and Health during Childhood: A Longitudinal Examination of Racial/Ethnic Differences in Parental Socioeconomic Timing and Child Obesity Risk

**DOI:** 10.3390/ijerph15040728

**Published:** 2018-04-11

**Authors:** Antwan Jones

**Affiliations:** Department of Sociology, The George Washington University, Washington, DC 20052, USA; antwan@gwu.edu; Tel.: +1-202-994-0266

**Keywords:** socioeconomic status, child health, obesity, overweight, race, ethnicity, parental influence, health disparities

## Abstract

Prior research suggests that socioeconomic standing during the early years of life, particularly in utero, is associated with child health. However, it is unclear whether socioeconomic benefits are only maximized at very young ages. Moreover, given the link between socioeconomic status (SES) and race, research is inconclusive whether any SES benefits during those younger ages would uniformly benefit all racial and ethnic groups. Using 1986–2014 data from the National Longitudinal Study of Youth (NLSY79), this study examines the impact of socioeconomic timing on child weight outcomes by race. Specifically, this research investigates whether specific points exist where socioeconomic investment would have higher returns on child health. Findings suggest that both the timing and the type of socioeconomic exposure is important to understanding child weight status. SES, particularly mother’s employment and father’s education, is important in determining child health, and each measure is linked to weight gain differently for White, Black, and Hispanic children at specific ages. Policies such as granting more educational access for men and work-family balance for women are discussed.

## 1. Introduction

Childhood development scholars have long argued that socioeconomic standing during the early years of life, particularly in utero [1], is associated with child health [2,3]. A child’s physical health at birth is most affected by the diminution of parental access to resources that buffer any negative effects of perinatal complications [4]. As a measure of physical health, child obesity is linked to the socioeconomic conditions within the family [5]. However, recent work has attempted to disentangle whether education, employment, and income uniformly translate to a benefit for all children at all ages [6]. Using a life-course perspective [7], studies have suggested that socioeconomic status (SES) operates similarly to compound interest: the effects of SES are greater the longer that a family maintains a particular level of SES [8,9]. In general, studies have not adequately explored how SES may interact with characteristics that are etiologic to its accrual, such as race and the age of the child. In fact, early work on SES and health suggests that SES differences in morbidity outcomes are actually class-based effects of health behaviors and knowledge [10,11]. Thus, characteristics associated with class, such as race or SES, are indirectly related to health. However, if class differences in health show themselves through race, then more research would find that SES effects would attenuate any racial differences in health. Yet, this is not the case: Race plays an independent role in predicting child health above and beyond any socioeconomic effects [12]. Therefore, it is key to inquire whether SES has a more robust effect in predicting child health for Whites than minorities. Moreover, since socioeconomic measures can vary over time, it is equally compelling to ask what role time has in clarifying the relationship between race, SES, and child health.

While SES effects on health are postulated to begin before birth [1,13], other research is couched in models that assert that there are distinct times after childbirth at which SES has its most crucial impact on health. The *childhood-limited model* suggests that the SES-health relationship is strongest in early childhood but then weakens with age [14]. If this model is correct, then early-life conditions are paramount in setting a child toward better health in later life. In this case, SES is tied to factors such as access to child care, parental attachment, and safe housing conditions [15,16]. The *adolescent-emergent model* suggests that the SES-health relationship is weak in early childhood but then strengthens with age [17]. In this model, factors such as peer influence or personality play a more resilient role. In fact, physical activity is more strongly correlated with SES during adolescence than earlier in childhood [18]. This model suggests that children begin to model the health behaviors of their parents early in life but as the child becomes older, the extent to which these behaviors are internalized and performed by the child will be more influential to child health. Thus, it is expected that SES effects on body mass index would be strongest in years where the child is making conscious decisions about physical activity and healthy eating, corresponding to later (rather than early) in the child’s life. The *persistence model* suggests that the SES-health relationship is constant across a child’s life course [19]. That is, factors that may influence child health or SES either do so constantly over time or have no impact over the child’s life course. For instance, the relationship between asthma severity and SES is no different in early childhood than in adolescence [18,20]. In this case, medical intervention may differentiate low- and high-SES households. Compared to high-SES families, low-SES families may have greater mistrust of the medical community, which, in turn, influences the adherence to instructions of medicine dosage and behaviors managing health [21]. Of course, medical mistrust is more pervasive in populations that have historically been victims of medical misconduct, namely racial/ethnic minorities and women [22,23]. Thus, prima facie evidence of a constant SES-health relationship may actually be persistent social class effects not captured by regular data sources.

Unequivocally, SES is paramount to positive child health outcomes. This study, which relies on longitudinal data for children who were biennially interviewed starting in 1986, investigates whether specific points exist in a child’s life where socioeconomic investment in the child will have higher returns on child health, and whether there may be racial/ethnic differences in those specific points. In short, at which age (or ages) in the life course does SES have the most protective impact, and does that age (or those ages) differ for White, Black, and Hispanic children?

## 2. Materials and Methods

### 2.1. Data

The National Longitudinal Survey of Youth (NLSY79) is a nationally representative sample of 12,686 young men and women who were 14–22 years old when they were first surveyed in 1979. The data are publicly available from the U.S. Bureau of Labor Statistics website, and its use in this study is not considered human subjects research, since it does not involve merging or enhancing data from the restricted data set where identifiers are present. Thus, no institutional review board approval is needed to use the publicly available data that this study utilizes.

Individuals in this survey were interviewed annually through 1994 and biennially from 1994 to 2014. Information on how the participants were selected are described elsewhere [24]. A key feature of this survey is that it gathers information in an event history format, in which dates are collected for the beginning and ending of important life events. On a regular basis, the NLSY79 has collected much pre- and post-natal care information from female respondents as they became mothers. These extensive data also include measures about the mother’s health during pregnancy and prenatal practices such as the extent of alcohol use or smoking and the use of prenatal care. Also, self-reported data on gestation length, birth weight, infant feeding practices, illnesses, and baby care during the first year of life are available.

In 1986, a separate survey of all children born to NLSY79 female respondents began, greatly expanding the breadth of child-specific information collected. In addition to all of the mother’s information from the NLSY79, the child supplement includes assessments of each child as well as additional demographic and developmental information collected from either the mother or child. For children younger than 10, many of the assessments and much of the supplemental information are collected from the child’s mother. For children aged 10 and older, information has been collected from the children biennially since 1986 on a variety of topics including child-parent interaction, attitudes toward schooling, dating and friendship patterns, religious attendance, health, substance use, and home responsibilities. The analyses presented herein relies on both samples (NLSY79 and the child supplement data), covering 30 years of interviews. The total number of person-period records (i.e., the analytic sample) is 124,265.

### 2.2. Dependent Measure

As a time-varying indicator of child health, body mass index (BMI) is calculated at each wave of data collection. Children (or mothers, when appropriate) are asked for their height (in inches) and their weight (in pounds). Alternatively, the interviewer may be given permission by the parent(s) to physically measure the child as well. Thus, this dependent variable combines self-reported, observed, and actual measurements of height and weight. Based on the child’s BMI, a percentile rank is given to each child that corresponds to medical growth charts provided by the Centers for Disease Control and Prevention [25]. To assess whether an *overweight transition* occurred, a dichotomous measure is calculated using the standard medical BMI percentile cut-off of 85%. When the child scores in the 85th percentile or higher, the child is considered to be developmentally overweight. If the child is below the 85th percentile, s/he is not considered to be overweight.

### 2.3. Parental Socioeconomic Measures

The socioeconomic condition of the household is measured at each wave using five independent variables that are derived from the main NLSY79 datafile. Mothers are asked for the highest grade or year of regular school that they have completed and received credit. In the original data, individuals who specify more than 20 years of education were given a value of 20. Thus, *mother’s years of education* corresponds to the highest grade completed on the interview date of the child. Information pertaining to the residential father (or father-figure) was also attained. *Father’s years of education* corresponds to the highest grade completed on the interview date of the child.

Mothers are asked how many weeks during the calendar year were they gainfully employed at their place of business. Thus, *mother’s weeks of employment* is a continuous measure that corresponds to the number of weeks (0–52) that the mother was employed. This measure does not include unpaid work (e.g., household labor or volunteer activities). Fathers were only asked if they were employed during the prior calendar year. As such, *employed father* is a dichotomous variable, indicating if the father reported working any time during the previous calendar year. Like mothers, the employment measure for fathers does not include unpaid work.

Family income is calculated from a series of questions that ask for specific dollar amounts received from various income sources. Those sources include income from wages, salaries, tips, unemployment compensation, business/farm income, child support, alimony, Aid to Families with Dependent Children (AFDC), Supplemental Security Income (SSI), disability, veteran benefits, and parental or relative support. Because respondents were to provide this information for the calendar year prior to the interview date, this measure is considered to be one-year lagged. *Family income* is thus the summed income of the mother and the father. To complete the measure, family income has been adjusted to 2014 dollars (the final year of data collection), according to the Consumer Price Index [26], an adjustment that will allow for cross-wave income comparisons. In the analytic models, a logged version of family income is used to lower the variance inflation factor (VIF) of the measure [27].

### 2.4. Maternal-Specific Measures

Maternal mechanisms centered around mother’s caretaking and demographic characteristics, which much research suggests is highly related to child obesity [28,29], are captured by several variables. Two dichotomous measures indicating whether the mother drank alcohol or smoked during a predefined amount of time are included. If the mother consumed any alcohol in the 12 months prior to the child’s birth, she is given a value of 1 for *drank alcohol*. All other mothers receive a 0. Likewise, if the mother smoked in the 12 months prior to the child’s birth, she is given a value of 1 for *smoked*. If the mother refrained from using tobacco, she received a 0. It is important to note here that, due to the imprecise nature of how the questions were asked to respondents, it is impossible to isolate poor maternal prenatal behaviors. *Mother breastfed* is a dichotomous variable that indicates whether the child was breastfed during the first years of his/her life through mother’s self-report.

*Mother’s age* is captured by self-report and is measured in years. For *mother’s BMI*, they were asked for their height (in inches) and their weight (in pounds). BMI is calculated using these two measurements [30]. Marital status is also crucial in understanding child health. For each wave of data collection, respondents were asked their marital status. These marital statuses are collapsed into *never married*, *married* (which includes those mothers who remarried), and *divorced* (which include mothers who were legally separated or widowed). The fourth marital category is calculated using the household roster. If mothers were not married (i.e., never married or divorced/separated/widowed) but listed a household member as their partner, they were considered to be *cohabiting*. This category also includes couples who never formally married, but may be in common law marriages.

### 2.5. Child Demographic Measures

Children’s demographic characteristics are also included. *Child’s age* is captured by self-report and is measured in years. Gender is also defined through self-report, with *males* being the comparison category. *First born* is a dichotomous variable that indicates whether the child was the first-born or only child. If the child weighed less than 5.5 pounds at birth, they are considered to be of *low birthweight*. Race is defined through self-report and is represented by three categories: *non-Hispanic White*; *non-Hispanic Black*; and *Hispanic*. Other racial categories could not be included due to the low sample size across the multiple waves of data.

### 2.6. Statistical Analyses

This research uses discrete-time event history analysis to assess the temporal, longitudinal effects of SES on child health. There are two groups of children that present selection issues in the data: children who were born prior to the first year of data collection (1986), and children who were born during the data collection but have not reached their 18th birthday at last wave (2014). For the first group, there is left truncation, and for the second group, there is right truncation. However, for the first group, selection was handled by using adjustments to delated entry bias, as seen in prior works [27,31,32]. To account for left censorship, children are given the same start time, which is the wave that they were eligible to be in the analytic sample (i.e., they were at least two years old). Thus “time” in this case corresponds to years followed in the survey.

Other manipulations of the data were also performed. Data for children who reached age 18 were not used, although the data prior to turning 18 remained. Since event history analysis tracks units over time until the units experience some defined event, children who were overweight at the start of their eligibility (i.e., they already experienced their overweight transition) were removed from the analytic sample. Individuals with missing values for the dependent measure were given a value of 0. This substitution assumes that any observed overweight transitions are the first transition experienced. Since SES is focal to this research, cases are excluded if the respondent had no valid measures for income, education, or employment for all waves of data. Mean substitution by wave of data is performed for covariates with missing values, where appropriate.

The model development used herein is additive. Model 1 uses variables for child’s age (age and age-squared). Model 2 adds children’s characteristics. Model 3 includes maternal mechanisms. Model 4 adds parental SES measures. In the final model, all SES-age interactions are entered. Lastly, race-stratified models are done separately to test how the predictors operate within racial groups. Following previous studies on child health transitions [33,34], this research uses the discrete model with a logit link since time in this research is discrete. The logit link function is easily implemented in statistical software, and it allows for direct interpretation in terms of conditional odds. Even though time is discrete, child’s age is used to specify the timing of overweight transitions. Odds ratio estimates are derived via maximum likelihood using Stata 14.2 (College Station, TX). Because multiple children are born to the same mother, observations are clustered according to the mother.

## 3. Results

Table 1 gives the unweighted descriptive statistics at baseline for the total sample and for each racial/ethnic group. Parental education does not vary widely across race/ethnicity, but employment and income measures have substantial variation. The average weeks of maternal employment at baseline for Whites is 31.3 weeks, compared to 24 weeks for Blacks and 25.1 weeks for Hispanics. For family income, White families on average start at $49,863 while Black families start at $30,422 and Hispanic families start at $39,798. The maternal mechanisms in this study vary greatly across race. While 51.5% of White mothers drank alcohol, only 32.7% of Blacks and 32.1% of Hispanics report that same behavior. Similarly, 38.3% of White mothers, 30.3% of Black mothers, and 18.6% of Hispanic mothers smoked. Similar percentages of White mothers (56.1%) and Hispanic mothers (50.2%) breastfed their child, but only 24.6% of Black mothers breastfed. While the vast majority of White mothers (61.8%) and Hispanic mothers (58.2%) were married at baseline, Black mothers were more represented in the data as never-married (48.8%). There are similar distributions across race with regards to the characteristics of the children at baseline, but there was a noticeable difference in the percentage of children who were born with low birthweight. Black children were born with low birth weight at over two times the rate as White children (14.4% vs. 7.1%). Approximately 8% of Hispanic children were born with low birthweight.

Table 2 shows the estimated Table 2 shows the estimated probabilities of children’s characteristics, maternal mechanisms, and SES on becoming overweight. Model 1, which acts as the time model, suggests that as children in the sample age, their probability of becoming overweight is reduced. Model 2 has the remaining child characteristics. Compared to White children, Black and Hispanic children have a greater probability of being overweight over time. Boys in the sample have a higher probability of becoming overweight than girls. However, being the only child or being the first born child is associated with a lower probability of becoming overweight than higher parity children in the household. In addition, a low birthweight is associated with a lower probability of becoming overweight.

Model 3 adds the maternal mechanisms to the previous model. The effect of being first born is attenuated, explained by the addition of mother’s age to this model (as auxiliary analyses revealed). Compared to mothers who refrained from drinking alcohol, mothers who did drink have a lower probability of the child becoming overweight over time. However, the reverse is found for smoking: mothers who smoked have a higher probability of the child becoming overweight over time relative to mothers who did not smoke. If the mother breastfed the child, the child would have a lower probability of becoming overweight than if she had not breastfed. Mother’s age increases the probability of becoming overweight, and somewhat axiomatic, mother’s obesity risk (proxied by BMI) elevates the probability that the child will become overweight. For marital status, being divorced is associated with a higher probability of child overweight compared to being married.

Model 4 is the full model with all covariates, including SES. From the previous model, two maternal mechanisms (drank alcohol and smoked) that were statistically significant are no longer significant. Auxiliary analyses indicate that the addition of all the SES variables contributed to this attenuation of effects. Net of the other controls, the only two SES variables that are significant are father’s years of education and mother’s weeks of employment, and their effects are moderately low. The more the father is educated, the lower the probability that the child will become overweight. Conversely, the more weeks the mother works, the higher the probability that the child will become overweight.

**Figure 1:**
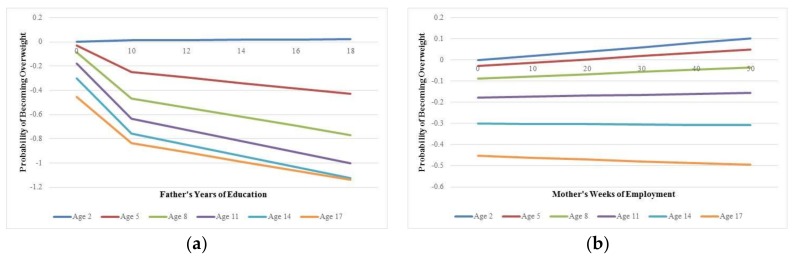
Visualization of the statistical interaction between parental socioeconomic status (SES) and child’s age in determining obesity risk: (**a**) Standardized probability of becoming overweight by father’s education and child’s age; (**b**) Standardized probability of becoming overweight by mother’s weeks of employment and child’s age.

Model 5 is the interaction model, where the two statistically significant SES measures (father’s education, mother’s employment) are interacted with the child age variables in order to uncover the point in a child’s life where SES has its greatest effect in predicting overweight. However, to illustrate this interaction effect, Figure 1 visualizes these interactions. In both panels, the *y*-axis shows the standardized probability of becoming overweight over time. For simplicity, the child ages that are represented as lines on the graph are: 2, 5, 8, 11, 14 and 17. Panel A shows the interaction of child’s age with father’s education. Here, there is little effect of becoming overweight across father’s educational attainment for children aged 2. However, as the age increases, there is a noticeable pattern: a steep drop in the probability of becoming overweight up until high school (father’s education = 10) and then a less steep drop as education level increases. Panel B shows the interaction of child’s age with mother’s weeks of employment. An interesting pattern emerges: Until age 11, mother’s work increases the probability of their child becoming obese the more she works. However, after age 11 (or particularly at age 17), mother’s work decreases the probability of becoming obese as the number of working weeks is increased.

Table 3 shows the estimated probabilities of children’s characteristics, maternal mechanisms, and SES on becoming overweight stratified by race/ethnicity. Two models are presented for each racial group: the main effects model, which has all covariates, and the interaction model, which has all covariates and the two statistically significant SES-age interactions. The table indicates that for some of the covariates, there are statistically significant effects that are consistent across race/ethnicity. However, the SES variables differ in effect by race. For White children, father’s years of education and mother’s weeks of employment are significant predictors of becoming overweight. For Black children, only mother’s weeks of employment is statistically related to the probability of becoming overweight. For Hispanic children, there was no relationship between SES and the probability of becoming overweight.

In the main effects model, there is a shift in direct of the relationship between mother’s weeks of employment and the probability of the child becoming overweight. The effect is positive for White children (i.e., the more weeks the mother works, the greater the probability of becoming overweight) but negative for Black children.

**Figure 2:**
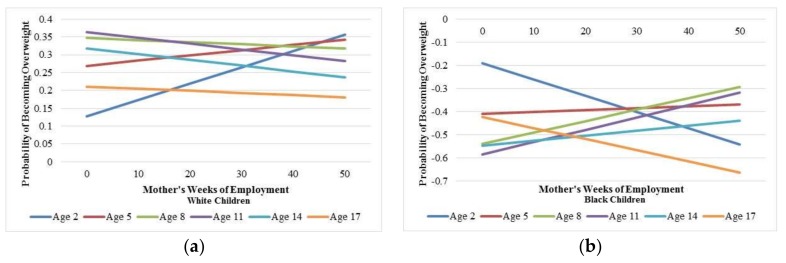
Visualization of the statistical interaction by race between mother’s weeks of employment and child’s age in determining obesity risk: (**a**) Unstandardized probability of becoming overweight by mother’s weeks of employment and child’s age for White children; (**b**) Unstandardized probability of becoming overweight by mother’s weeks of employment and child’s age for Black children.

In order to tease out these complex racial effects, the interaction model findings were graphed. Figure 2 has a side-by-side comparison of the effects of mother’s weeks of employment by age for Whites (Panel A) and Blacks (Panel B) in predicting the probability of becoming overweight. The probabilities are not standardized, so it is impossible to determine the effect sizes using these figures. For White children, at very young ages (age 2), the more weeks the mother works, the higher the probability of becoming obese. However, for middle and late ages (ages 5+), mother’s employment lowers the probability of becoming obese. For Black children, at both very young (age 2) and very old (age 17) ages, the more weeks the mother works, the lower the probability of becoming obese. But, for Black children in between the ages of 2 and 17, mother’s employment elevates obesity risk.

## 4. Discussion

This study provides evidence that the timing of socioeconomic exposure is as important to understanding child weight status as the type of socioeconomic exposure. This research confirmed that SES matters, particularly mother’s employment and father’s education. Prior work suggests that mother’s employment may have an effect on the nutritional habits of their children and the nutrition knowledge that the children may learn from their parents. Working parents generally have less time available to cook and prepare meals [35], less energy to supervise and participate in their children’s eating patterns [36], and more household income that may provide disposable income to either eat better quality food or restaurant meals more often [37,38]. Interestingly, the effect for employment is only seen for mothers and not fathers. The limited father-specific socioeconomic measures may have contributed to the statistically non-significant effect of father’s employment in child obesity risk. Future research should attempt to provide and analyze more comprehensive socioeconomic measures for all parents and guardians in the household.

In the reverse, father’s education, not mother’s education, significantly predicted child weight. Prior work on education and child health has focused on mother’s education, citing that there may be endogeneity between mother’s and father’s education because spouses tend to be equally educated [39]. In this sample, at baseline, there seems to be some concordance in the average years of education for mothers and fathers. However, auxiliary analyses performed indicate that the correlation between the two is only moderate across all 30 years (the highest correlation was 0.35), suggesting a unique contribution of father’s education in predicting child weight. Studies suggest that education itself is not directly related to child health, but rather, the underlying and unobserved characteristics tied to education such as parental health, parental income during childhood and community access to programs operate through parental education [40]. However, with few studies that include education measures for both parents, future studies should consider gender-specific pathways that could make father’s education as important of a predictor as mother’s educational attainment.

This research explored some of the underlying characteristics tied to SES by adjusting for maternal mechanisms to child weight. The study documents that the behavior of the mother does matter to child obesity risk. Mother’s body mass index, a proxy for weight status, is related to their child’s weight. Older mothers also tended to have children at higher chances of becoming overweight thank younger mothers. In addition, breastfeeding, which is tied to SES [41,42,43], lowered the child’s risk of becoming overweight, although this effect is primarily seen in White children. Prior research suggests that bottle-fed children are more likely to be overweight as children and adults than children who breastfed [44,45], and working mothers are generally less able to breastfeed or they may stop breastfeeding at an earlier age than mothers who do not work [46].

While there is a racial difference in the effect of one maternal mechanism on child weight status, a more complex relationship between race, the timing of socioeconomic exposure, and overweight risk emerged from this study. Race and SES are very much tied in the US, such that racial differences in health are similar to SES differences in health. Generally, father’s education and mother’s weeks of employment had its greatest impact at different ages. For father’s education, when the child is five years old is where we start to see the start of cumulative gains in reducing overweight risk, particularly when the father has completed at least some high school. For mother’s weeks of employment, this research found that around age 14, the potential negative consequences of outside-of-home work for mothers is considerably decreased. However, because each of the SES-child’s age operate differently, this research must conclude that socioeconomic effects benefit children at different ages depending on which SES measure is used. During the formative years of child development, income is very important, since it creates material conditions to ensure healthy development. Families can afford the healthy maintenance of the child’s health through selection of foods and access to medical care. However, as the child ages, the knowledge accrued from both parents through formal education is critical to child development. Parents who are highly educated may be more proactive in their child’s health supervising proper nutrition, selecting low-fat snacks, maintaining good exercise/activity habits, and monitoring the television viewing of their children. Highly educated parents may also focus on building self-esteem to help address psychological issues of their children, which could cause negative health behaviors.

Even so, the ages that these socioeconomic measures have their greatest impact differ among White and Black children. For White children, mother’s work outside of the home before age 8 seems to be its most detrimental to child overweight risk. However, for Black children, it is at both younger (age 2) and older (age 17) ages where working outside of the home confers its highest benefit. It is also noteworthy that in this study, there was no socioeconomic effect for Hispanic children in predicting the chances that the child would be overweight. Collectively, all show that SES does not operate similarly within racial groups; some SES measures are considered to be beneficial to particular racial groups and other SES measures are not. To date, no studies have explored racial differences in exposure of socioeconomic timing as it relates to child obesity risk. However, research on racial and socioeconomic differences in child obesity suggests that in addition to the lower SES that minorities general have compared to Whites, the cultural differences in parenting, health behaviors, and acquisition/maintenance of SES may be mechanisms that help uncover what is driving racial differences in child health [47]. Due to participant attrition and statistical power concerns, important racial and ethnic group comparisons could not be explored. Future research should also attempt to look at other marginalized groups in contrast to the groups presented here as well as separate entities to understand cultural experiences affecting the SES-health relationships. As such, Native Americans, various Asian Americans groups and more diversification of Black (e.g., Afro-Caribbeans, Africans) and Hispanic (e.g., Puerto Ricans, Mexicans) groups would further the research on race, parental SES, and child health.

Results from this study illuminate the disparate impact that some measures of SES have in determining child health. However, due to data restrictions, other measures of SES could not be used. Thus, future research on this topic should conceptualize SES in all possible ways, including the ones that were not used in this study. While there is an issue with the measurement of wealth across waves in the NLSY, we know that wealth is an important and independent predictor of child health [48], and its inclusion would have likely yielded a different perspective of how SES influences obesity risk among children. Moreover, while objective measures of SES proved to be statistical predictors to child health, subjective measures (particularly from the children) would have been beneficial to use since both could validate the objective SES measures and provide a different understanding of what social class the child perceives the family to be. In addition, using SES measures external to the individual such as community or spatial SES [49] would also be useful in underscoring how SES at all levels affects the health and well-being of children. This study informs one aspect of causation being the possibility that SES is a factor that predicts obesity transitions, but this study does not lead to conclusions that SES is causally linked to obesity. Although the vibrant data come from 30 years of observation, it cannot overcome the limitation that it is secondary data and thus cannot infer causal inference.

Several results of this study are policy-relevant and command future research attention to their policy implications. First, education, particularly the father’s, seems to be highly explanatory of childhood BMI. This suggests that increasing paternal education can help curb the child’s risk of being overweight. Future research should explore how programs designed to help fathers complete high school or college can impact their children’s health. One of the major public health endeavors has been to fuse the exercise needs of a sedentary childhood with the nutritional needs of the child during school hours in order to curb childhood obesity. While this research could not include variables that measure lifestyle behaviors among children, it would be fruitful for future research to explore how SES enables children to engage in these healthy behaviors.

## 5. Conclusions

Mother’s work outside of the home was most detrimental to child weight before the age of eight for White children. For Black children, around ages two and 17, mother’s work was the most beneficial to curbing child obesity risk. Mother’s work had no effect on weight for Hispanic children. The effect of father’s education was constant across race—starting at age five, father’s education buffered their child’s obesity risk, especially when the father has at least completed high school. While these race and socioeconomic effects have been widely discussed in prior child health research, this study’s unique contribution is the examination of the age- and race-specific effects of maternal and paternal socioeconomic measures on child obesity risk.

## Figures and Tables

**Table 1:** Unweighted Baseline Characteristics by Race/Ethnicity.

	Total		Non-Hispanic White		Non-Hispanic Black		Hispanic	
Baseline Characteristics	Mean or % (SE)		Mean or % (SE)		Mean or % (SE)		Mean or % (SE)	
SES Variables								
Mother’s Years of Education ^‡^	12.23	(2.29)	12.59	−2.26	12.17	−1.83	11.32	−2.68
Father’s Years of Education ^‡^	12.38	(2.41)	12.74	(2.20)	12.50	(1.83)	11.16	(2.97)
Mother’s Weeks of Employment ^‡^	28.10	(22.10)	31.34	(21.53)	23.96	(22.20)	25.13	(22.09)
Employed Father ^‡^	94.98%	---	95.77%	---	93.26%	---	94.09%	---
Family Income ^‡^	$42,824.46	−34,925.45	$49,862.51	−36,505.07	$30,422.91	−27,503.58	$39,797.63	−34,287.65
Maternal Mechanisms								
Drank Alcohol	42.78%	---	51.51%	---	32.74%	---	32.07%	---
Smoked	32.45%	---	38.32%	---	30.25%	---	18.58%	---
Mother Breastfed	46.34%	---	56.05%	---	24.64%	---	50.21%	---
Mother’s Age	25.41	(5.98)	25.84	−5.78	24.37	−6.11	25.73	−6.19
Mother’s BMI ^‡^	23.71	(4.71)	22.91	(4.37)	24.80	(5.11)	24.35	(4.58)
Marital Status ^‡^								
Never Married	28.48%	---	19.62%	---	48.79%	---	23.40%	---
Married	52.91%	---	61.80%	---	32.39%	---	58.23%	---
Cohabiting	7.42%	---	8.17%	---	6.30%	---	6.99%	---
Divorced	11.19%	---	10.42%	---	12.52%	---	11.38%	---
Children’s Characteristics								
Child’s Age (in years) ^‡^	5.99	(3.51)	6.00	(3.61)	6.20	(3.41)	5.63	(3.35)
Male	51.09%	---	50.94%	---	51.11%	---	51.47%	---
First born	42.42%	---	45.84%	---	39.30%	---	37.31%	---
Low Birthweight	9.30%	---	7.10%	---	14.43%	---	8.07%	---

**S**ource: National Longitudinal Surveys. Note: Percentages may not add up to 100% due to rounding. ^‡^ indicates a time-varying variable.

**Table 2:** Multivariate Adjusted PROBIT of Becoming Overweight.

	Model 1		Model 2		Model 3		Model 4		Model 5	
**Children’s Characteristics**										
Child’s Age	−0.15	***	−0.15	***	−0.14	***	−0.14	***	0.00	
	−0.01		−0.01		−0.01		−0.01		−0.05	
Child’s Age^2^	0.00	***	0.00	***	0.00	***	0.00	***	0.00	
	−0.00		−0.00		−0.00		−0.00		−0.00	
Race *(White)*										
Black			0.24	***	0.16	***	0.15	***	0.15	***
			−0.02		−0.03		−0.04		−0.04	
Hispanic			0.20	***	0.17	***	0.14	***	0.14	***
			−0.02		−0.03		−0.04		−0.04	
Male			0.05	**	0.06	**	0.10	***	0.11	***
			−0.02		−0.02		−0.03		−0.03	
First born			−0.06	**	0.03		0.01		0.01	
			−0.02		−0.02		−0.03		−0.03	
Low Birthweight			−0.08	**	−0.07	*	−0.12	**	−0.12	*
			−0.03		−0.04		−0.05		−0.05	
**Maternal Mechanisms**										
Drank Alcohol					−0.08	***	−0.05		−0.06	
					−0.02		−0.03		−0.03	
Smoked					0.10	***	0.06		0.06	
					−0.02		−0.03		−0.03	
Mother Breastfed					−0.07	**	−0.07	**	−0.07	**
					−0.02		−0.03		−0.03	
Mother’s Age					0.03	***	0.03	***	0.03	***
					−0.00		−0.00		−0.00	
Mother’s BMI					0.03	***	0.02	***	0.02	***
					−0.00		−0.00		−0.00	
Marital Status *(Married)*										
Never Married					0.02		0.00		0.00	
					−0.03		−0.00		−0.00	
Cohabiting					0.04		0.07		0.07	
					−0.04		−0.05		−0.05	
Divorced					0.07	*	0.06	*	0.04	
					−0.03		−0.02		−0.02	
SES Variables										
Mother’s Years of Education							0.00		0.00	
							−0.01		−0.01	
Father’s Years of Education							−0.02	***	0.02	
							−0.01		−0.02	
Mother’s Weeks of Employment							0.00	*	0.00	
							−0.00		−0.00	
Employed Father							−0.03		−0.03	
							−0.06		−0.06	
Log Family Income							0.02		0.02	
							−0.01		−0.01	
**SES-Child’s Age Interactions**										
Father’s Years of Education x Child’s Age									−0.01	***
									−0.00	
Mother’s Weeks of Employment x Child’s Age									0.00	*
									−0.00	
**SES-Child’s Age^2^ Interactions**										
Father’s Years of Education x Child’s Age^2^									0.00	*
									−0.00	
Mother’s Weeks of Employment x Child’s Age^2^									0.00	
									−0.00	
Constant	−0.55	***	−0.67	***	−2.14	***	−2.01	***	−2.62	***
	−0.03		−0.03		−0.09		−0.17		−0.25	
Wald χ^2^	2029.28	***	1894.56	***	1824.10	***	1085.10	***	1091.09	***
-2 Log Pseudolikelihood [-2LL]	25,337.39		22,395.51		19,312.78		11,436.25		11,419.32	
Pseudo R-square	0.08		0.08		0.10		0.10		0.10	
Deviance Statistic ^‡^	----		2941.89	***	3082.73	***	7876.53	***	16.94	***

**S**ource: National Longitudinal Surveys. Note: Contrast categories are in parentheses *** *p* < 0.001; ** *p* < 0.01; * *p* < 0.05. ^‡^ Deviance statistic is the difference in the current model and the previous model’s (i.e., saturated model) -2 LL (Singer and Willett 2003).

**Table 3:** Multivariate Adjusted PROBIT of Becoming Overweight by Race/Ethnicity.

	Non-Hispanic White				Non-Hispanic Black				Hispanic			
	Main Effects Model		Interaction Model		Main Effects Model		Interaction Model		Main Effects Model		Interaction Model	
Children’s Characteristics												
Child’s Age	−0.15	***	0.07		−0.11	***	−0.11		−0.15	***	0.00	
	−0.02		−0.08		−0.03		−0.17		−0.03		−0.08	
Child’s Age^2^	0.00	***	0.00		0.00		0.00		0.00	*	0.00	
	−0.00		−0.00		−0.00		−0.01		−0.00		−0.00	
Male	0.11	***	0.11	***	0.09		0.10		0.11	*	0.11	*
	−0.03		−0.03		−0.06		−0.06		−0.06		−0.06	
First born	0.00		0.00		0.06		0.06		0.01		0.01	
	−0.04		−0.04		−0.07		−0.07		−0.06		−0.06	
Low Birthweight	−0.20	**	−0.20	**	0.08		0.07		−0.21	*	−0.21	
	−0.07		0.07		−0.09		−0.09		−0.12		−0.12	
Maternal Mechanisms												
Drank Alcohol	−0.04		−0.04		−0.11		−0.11		−0.04		−0.04	
	−0.04		−0.04		−0.07		−0.07		−0.06		−0.06	
Smoked	0.04		0.04		0.09		0.09		0.01		0.02	
	−0.04		−0.04		−0.08		−0.08		−0.08		−0.08	
Mother Breastfed	−0.10	**	−0.09	*	0.03		0.02		−0.08		−0.09	
	−0.04		−0.04		−0.07		−0.07		−0.06		−0.06	
Mother’s Age	0.03	***	0.04	***	0.03	***	0.03	***	0.03	***	0.03	***
	−0.00		−0.00		−0.01		−0.01		−0.01		−0.01	
Mother’s BMI	0.02	***	0.02	***	0.03	***	0.03	***	0.02	***	0.02	***
	−0.00		−0.00		−0.01		−0.01		−0.01		−0.01	
Marital Status (*Married*)												
Never Married	0.00		0.00		0.00		0.00		0.00		0.00	
	−0.00		−0.00		−0.00		−0.00		−0.00		−0.00	
Cohabiting	0.16	*	0.16	*	−0.04		−0.04		0.08		0.08	
	−0.07		−0.07		−0.09		−0.09		−0.09		−0.09	
Divorced	−0.01		−0.03		0.51		0.05		0.06		0.06	
	−0.05		−0.05		−0.31		−0.03		−0.04		−0.04	
SES Variables												
Mother’s Years of Education	0.00		0.00		0.00		0.00		0.01		0.01	
	−0.01		−0.01		−0.02		−0.02		−0.01		−0.01	
Father’s Years of Education	−0.04	***	0.02		0.00		0.05		−0.01		0.04	
	−0.01		−0.02		−0.02		−0.05		−0.01		−0.03	
Mother’s Weeks of Employment	0.01	**	0.01	**	−0.01	***	−0.01	***	0.00		0.00	
	−0.00		−0.00		−0.00		−0.00		−0.00		−0.00	
Employed Father	−0.02		0.00		0.00		0.00		−0.09		−0.10	
	−0.08		−0.08		−0.12		−0.12		−0.12		−0.12	
Log Family Income	0.03		0.03		0.02		0.02		0.00		0.00	
	−0.02		−0.02		−0.03		−0.03		−0.02		−0.02	
SES-Child’s Age Interactions												
Father’s Years of Education x Child’s Age			−0.01	*			−0.01				−0.01	
			−0.01				−0.01				−0.01	
Mother’s Weeks of Employment x Child’s Age			0.00	*			0.00	**			0.00	
			−0.00				−0.00				−0.00	
SES-Child’s Age^2^ Interactions												
Father's Years of Education x Child’s Age^2^			0.00				0.00				0.00	
			−0.00				−0.00				−0.00	
Mother’s Weeks of Employment x Child’s Age^2^			0.00				0.00	**			0.00	
			−0.00				−0.00				−0.00	
Constant	−2.00	***	−2.96	***	−2.52	***	−2.79	**	−1.64	***	−2.22	***
	−0.25		−0.37		−0.42		−0.76		−0.32		−0.43	
Wald χ^2^	631.15	***	632.06	***	203.54	***	217.13	***	253.71	***	256.22	***
-2 Log Pseudolikelihood [-2LL]	6740.86		6715.61		2195.28		2182.98		2467.13		2462.06	

**S**ource: National Longitudinal Surveys. Note: Contrast categories are in parentheses. *** *p* < 0.001; ** *p* < 0.01; * *p* < 0.05. A Chow test (Demaris 2004) performed indicated that the race-SES interaction terms would not be a statistically significant addition to the model (χ^2^(40) = 51.36).

## References

[1] Antonovsky A., Bernstein J. (1977). Social class and infant mortality. Soc. Sci. Med..

[2] Egbuonu L., Starfield B. (1982). Child health and social status. Pediatrics.

[3] Aber J.L., Bennett N.G., Conley D.C., Li J. (1997). The effects of poverty on child health and development. Ann. Rev. Public Health.

[4] McLoyd V.C. (1998). Socioeconomic disadvantage and child development. Am. Psychol..

[5] Jones A. (2016). Intergenerational educational attainment, family characteristics and child obesity. J. Biosoc. Sci..

[6] Power C., Manor O., Matthews S. (1999). The duration and timing of exposure: Effects of socioeconomic environment on adult health. Am. J. Public Health.

[7] Elder G.H., Rockwell R.C. (1979). The life-course and human development: An ecological perspective. Int. J. Behav. Dev..

[8] Currie J., Stabile M. (2003). Socioeconomic status and child health: Why is the relationship stronger for older children?. Am. Econ. Rev..

[9] Willson A.E., Shuey K.M., Elder J., Glen H. (2007). Cumulative advantage processes as mechanisms of inequality in life course health. Am. J. Sociol..

[10] James G. (1965). Poverty and public health—New outlooks: I. Poverty as an obstacle to health progress in our cities. Am. J. Public Health Nations Health.

[11] Taylor L. (1975). Poverty, wealth, and health, or getting the dosage right. Br. Med. J..

[12] Kawachi I., Daniels N., Robinson D.E. (2005). Health disparities by race and class: Why both matter. Health Aff..

[13] Gluckman P.D., Hanson M.A., Cooper C., Thornburg K.L. (2008). Effect of in utero and early-life conditions on adult health and disease. N. Engl. J. Med..

[14] Spencer N.J. (2006). Social equalization in youth: Evidence from a cross-sectional british survey. Eur. J. Public Health.

[15] Chen E. (2004). Why socioeconomic status affects the health of children: A psychosocial perspective. Curr. Dir. Psychol. Sci..

[16] Currie J., Hyson R. (1999). Is the impact of health shocks cushioned by socioeconomic status? The case of low birthweight. Am. Econ. Rev..

[17] Levin K.A., Currie C., Muldoon J. (2009). Mental well-being and subjective health of 11-to 15-year-old boys and girls in scotland, 1994–2006. Eur. J. Public Health.

[18] Chen E., Matthews K.A., Boyce W.T. (2002). Socioeconomic differences in children’s health: How and why do these relationships change with age?. Psychol. Bull..

[19] Hungerford T.L. (2007). The persistence of hardship over the life course. Res. Aging.

[20] Mielck A., Reitmeir P., Wjst M. (1996). Severity of childhood asthma by socioeconomic status. Int. J. Epidemiol..

[21] Born W., Engelman K., Greiner K.A., Bhattacharya S.B., Hall S., Hou Q., Ahluwalia J.S. (2009). Colorectal cancer screening, perceived discrimination, and low-income and trust in doctors: A survey of minority patients. BMC Public Health.

[22] Sheppard V.B., Zambrana R.E., O’malley A.S. (2004). Providing health care to low-income women: A matter of trust. Fam. Pract..

[23] Corbie-Smith G., Thomas S.B., St. George D.M.M. (2002). Distrust, race, and research. Arch. Intern. Med..

[24] U.S. Bureau of Labor Statistics The nlsy79 Sample: An Introduction. https://www.nlsinfo.org/content/cohorts/nlsy79/intro-to-the-sample/nlsy79-sample-introduction.

[25] Ogden C.L., Kuczmarski R.J., Flegal K.M., Mei Z., Guo S., Wei R., Grummer-Strawn L.M., Curtin L.R., Roche A.F., Johnson C.L. (2002). Centers for disease control and prevention 2000 growth charts for the united states: Improvements to the 1977 national center for health statistics version. Pediatrics.

[26] US Bureau of Labor Statistics Cpi Inflation Calculator. https://data.bls.gov/cgi-bin/cpicalc.pl.

[27] DeMaris A. (2004). Regression with Social Data: Modeling Continuous and Limited Response Variables.

[28] Fertig A., Glomm G., Tchernis R. (2009). The connection between maternal employment and childhood obesity: Inspecting the mechanisms. Rev. Econ. Househ..

[29] Morrissey T.W. (2013). Trajectories of growth in body mass index across childhood: Associations with maternal and paternal employment. Soc. Sci. Med..

[30] Centers for Disease Control and Prevention Calculating Bmi Using the English System. https://www.cdc.gov/nccdphp/dnpao/growthcharts/training/bmiage/page5_2.html.

[31] Cleves M. (2008). An Introduction to Survival Analysis Using Stata.

[32] Matsuura M., Eguchi S. (2005). Modeling late entry bias in survival analysis. Biometrics.

[33] Gunderson E.P., Quesenberry C.P., Lewis C.E., Tsai A.L., Sternfeld B., West D.S., Sidney S. (2004). Development of overweight associated with childbearing depends on smoking habit: The coronary artery risk development in young adults (cardia) study. Obesity.

[34] Smolak L., Smolak L., Levine M.P., Striegel-Moore R. (1996). Methodological implications of a developmental psychopathology approach to the study of eating problems. The Developmental Psychopathology of Eating Disorders: Implications for Research, Prevention, and Treatment.

[35] Fulkerson J.A., Kubik M.Y., Rydell S., Boutelle K.N., Garwick A., Story M., Neumark-Sztainer D., Dudovitz B. (2011). Focus groups with working parents of school-aged children: What’s needed to improve family meals?. J. Nutr. Educ. Behav..

[36] Morin P., Demers K., Turcotte S., Mongeau L. (2013). Association between perceived self-efficacy related to meal management and food coping strategies among working parents with preschool children. Appetite.

[37] West D.A., Price D.W. (1976). The effects of income, assets, food programs, and household size on food consumption. Am. J. Agric. Econ..

[38] Hymans S.H., Shapiro H.T. (1976). The allocation of household income to food consumption. J. Econom..

[39] Rosenfeld M.J. (2008). Racial, educational and religious endogamy in the united states: A comparative historical perspective. Soc. Forces.

[40] Thomas D., Strauss J., Henriques M.-H. (1991). How does mother’s education affect child height?. J. Hum. Resour..

[41] Flacking R., Nyqvist K.H., Ewald U. (2007). Effects of socioeconomic status on breastfeeding duration in mothers of preterm and term infants. Eur. J. Public Health.

[42] Amir L.H., Donath S.M. (2008). Socioeconomic status and rates of breastfeeding in australia: Evidence from three recent national health surveys. Med. J. Aust..

[43] De Barros F.C., Victora C.G., Vaughan J.P. (1986). Breastfeeding and socioeconomic status in southern brazil. Acta Paediatr..

[44] Butte N.F. (2001). The role of breastfeeding in obesity. Pediatr. Clin..

[45] Bartok C.J., Ventura A.K. (2009). Mechanisms underlying the association between breastfeeding and obesity. Pediatr. Obes..

[46] Yilmaz G., Gürakan B., Akgün S., Ozbek N. (2002). Factors influencing breastfeeding for working mothers. Turk. J. Pediatr..

[47] Williams D.R., Collins C. (1995). Us socioeconomic and racial differences in health: Patterns and explanations. Ann. Rev. Sociol..

[48] Meer J., Miller D.L., Rosen H.S. (2003). Exploring the health-wealth nexus. J. Health Econ..

[49] Fotso J.-C., Kuate-Defo B. (2005). Socioeconomic inequalities in early childhood malnutrition and morbidity: Modification of the household-level effects by the community ses. Health Place.

